# On the function of the ultimate legs of some Scolopendridae (Chilopoda, Scolopendromorpha)

**DOI:** 10.3897/zookeys.510.8674

**Published:** 2015-06-30

**Authors:** Christian Kronmüller, John G. E. Lewis

**Affiliations:** 1 Bavarian State Collection of Zoology, Münchhausenstr. 21, D-81247 Munich; 2 Manor Mill Farm, Halse, Taunton, Somerset TA4 3AQ, UK

**Keywords:** Chilopoda, Scolopendromorpha, ultimate legs, prey capture, defence reaction, courtship behaviour, mating

## Abstract

The function of the variously shaped ultimate legs of Scolopendridae is briefly reviewed. Their function in *Scolopendra heros* Girard, 1853, *Scolopendra subspinipes* Leach, 1815, *Scolopendra morsitans* (Linnaeus, 1758), *Scolopendra galapagoensis* Bollman, 1889, *Scolopendra hainanum* Kronmüller, 2012, *Scolopendra spinosissima* Kraepelin, 1903 *Cormocephalus aurantiipes* (Newport, 1844) and *Ethmostigmus trigonopodus* (Leach, 1817), in which they are least specialised has been investigated. Specimens were tapped with forceps on different parts of the trunk to simulate the attack of a predator. When tapped on the first third of the trunk (near the head), the centipedes attacked the forceps with their forcipules. When tapped on the last third or the ultimate legs, they adopted a warning position, raising the ultimate legs to display the ventral and medial prefemoral spines as well as the spined coxopleural processes. In some cases the centipedes attacked the forceps with the claws of the ultimate legs by chopping down on them after lifting the legs high into the warning position. When tapped in the mid part of the trunk, the centipedes curled sideways to reach the forceps with their forcipules and ultimate legs simultaneously. *Scolopendra galapagoensis* not only lifted the ultimate legs into the warning position but also the last 3-4 pairs of locomotory legs, presenting their distodorsal prefemoral spines. This resembles the warning posture of some spiders. In addition to their function in warning behaviour, defensive stabbing, ritualised meeting reactions and during courtship behaviour, the ultimate legs may in addition act as hooks and perhaps be involved in species recognition. No evidence was found that the ultimate legs are used to catch prey, nor of prey or predators being held between the prefemora.

## Introduction

**Figure 1.**
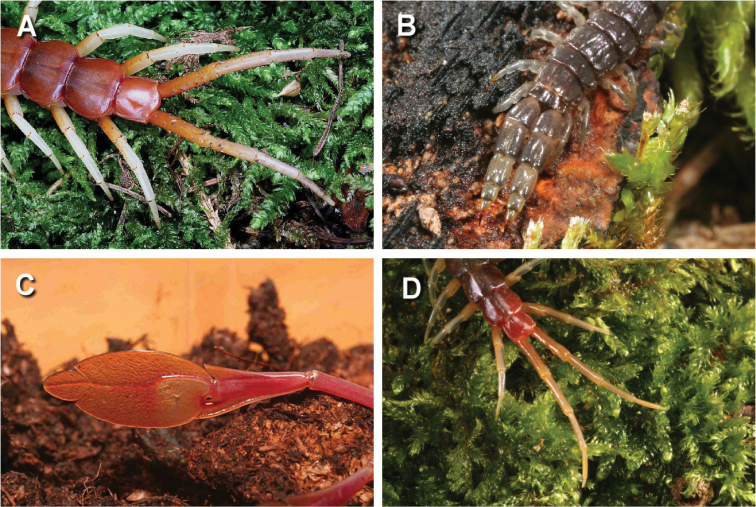
Different shape of ultimate legs: **A** The least specialised ultimate legs of *Scolopendra gigantea* **B** pincer-like ultimate legs in *Asanada akashii* **C** leaf-like ultimate legs of *Alipes grandidieri* **D** the long and slender ultimate legs of *Rhysida longipes* that can be autotomised.

The ultimate legs of scolopendrids exhibit a variety of shapes, the majority being what Schileyko (2009) called the common shape, the least specialised and most like locomotory legs (Figure 1A). They are pincer-shaped in *Scolopendropsis*, *Edentistoma*, and *Arthrorhabdus* and this type Schileyko stated are adapted for capturing prey (Figure 1B). The African scolopendrid *Asanada socotrana* Pocock, 1899, also has pincer-like legs. These lack prefemoral spines and are probably involved not in the capture of prey but in the distraction of a would-be predator as they are readily autotomised and when detached perform wriggling movements (Lewis, 1981). Species of the genus *Alipes* have large leaf-like ultimate legs (Figure 1C). *Alipes grandidieri* Lucas, 1864 when irritated swings these from side to side and stridulates perhaps to frighten potential mammalian predators (Skovmand and Enghoff 1980). The legs are sometimes autotomised and when this happens the legs continue to stridulate. Cloudsley-Thompson (1961) reported that the long ultimate legs of the West African *Rhysida nuda togoensis* Kraepelin, 1903 (now *Rhysida immarginata togoensis*) (Figure 1D) slowly bend and straighten when detached and emit a faint creaking sound.

In this paper we review the functions of ultimate legs in those species that have the least modified ultimate legs and report the results of laboratory experiments on species of *Scolopendra*, *Cormocephalus* and *Ethmostigmus*.

## Review of the function of the ultimate legs in the Scolopendridae

### Anchoring

Field observations have shown that the ultimate legs of scolopendrids are used as hooks from which the animal can hang. Thus Remington (1950) reported that *Scolopendra subspinipes* Leach, 1815, fastened onto a tent near the ventilator hole with their ultimate legs and swung their bodies to one side or the other to seize insects that had alighted nearby. On 4.xi.95 Peter Daszak and Janet Cottingham found a female *Scolopendra abnormis* Lewis & Daszak, 1996, hanging by her ultimate legs in a hollowed out cavity beneath a slab of tuff on Serpent Island, Mauritius. The anterior part of the body was curled around 40 oval yellow eggs (Lewis unpublished data).

Molinari et al (2005) observed two specimens of *Scolopendra gigantea* L., 1758, feeding on bats whilst hanging from the roof of a Venezuelan cave to which they anchored using the last five or eight pairs of legs.

Kronmüller (2013, unpublished data) reported and photographed a *Scolopendra dehaani* that escaped the plastic container in the laboratory and was later found hanging on the camera tripod just using the ultimate legs (Figure 3A).

### Defence reactions

**Warning posture**

Lewis (unpublished data) noted in Nigeria that a small *Scolopendra morsitans* L., 1758, raised and splayed its ultimate legs when approached from behind by a large *Mecistocephalus* (Geophilomorpha). Neck (1985) described such threat display in *Scolopendra viridis* Gervais, 1847, the ultimate legs being spread apart and in *Scolopendra heros* Girard, 1853, where the legs were raised and waved back and forth, active defence involving biting or “holding” with the ultimate legs. A photo of an *Ethmostigmus* sp. sent to one of us (J G E L) by Chris Pennington from Krambach, New South Wales, Australia, showed the ultimate legs lifted and splayed revealing the spines on the median surface of the prefemora and on the coxopleural processes. A predator approaching from behind would come into contact with a battery of spines.

**Autotomy and sound production**

As noted above, in case of danger the members of the genus *Alipes* swing the ultimate legs from side to side and stridulate perhaps to distract or frighten potential predators.. They also use autotomy to detract potential predators like birds, lizards or mammals. In case of an attack, the centipede starts to stridulate which directs the attention of the predator to the ultimate legs which can be autotomised to make the escape easier. When detached with forceps the leaf-like ultimate legs of an *Alipes grandidieri* from Tanzania continued to stridulate for more than half a minute.

Cloudsley-Thompson’s (1961) observed that the long ultimate legs of the West African *Rhysida nuda togoensis* (now *Rhysida immarginata togoensis*) slowly bend and straighten when detached and emit a faint creaking sound. This observations were repeated in Northern Nigeria in 1967 by Lewis (unpublished data): an ultimate leg of the same species detached with forceps produced very quiet squeaking for about 75 seconds, flexing at 2–3 second intervals and Kronmüller (2009, unpublished data) observed that the long slender ultimate legs of *Rhysida immarginata immarginata* (Porat, 1876) from the Philippines (Figure 1D) as well as *Rhysida longipes* (Newport, 1845) from various countries in Africa bend and straighten for a few minutes presumably to enable the centipede to flee while potential predators become distracted. In our experiments, when the ultimate legs were detached with forceps,they did not squeak as did those of *Rhysida immarginata togoensis*.

Video clips of *Alipes* sp. showing the typical defence behaviour including the sound of the stridulation as well as the bending movements of the detached ultimate legs of *Rhysida* sp. can be seen on the website http://www.scolopendromorpha.com.

## Intraspecific reactions

**Meeting reactions**

**Figure 2.**
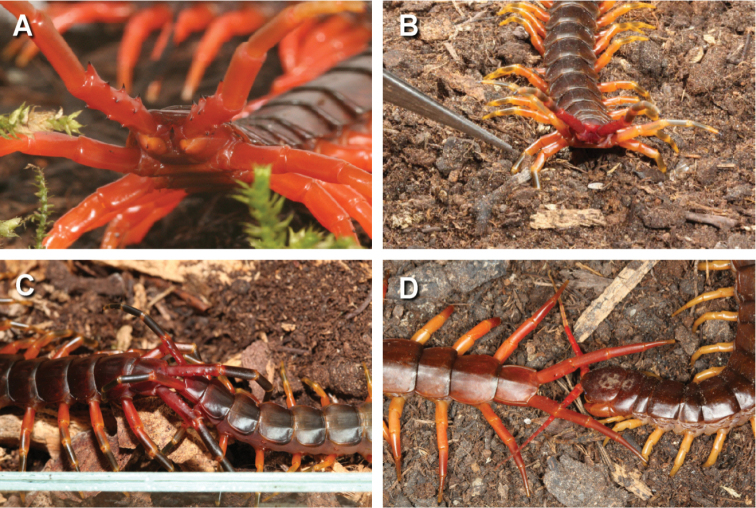
**A** Warning posture of *Scolopendra spinosissima* **B** Warning posture with lifted ultimate legs as well as the last pairs of locomotory legs in *Scolopendra galapagoensis* **C** Interlocked ultimate legs in a pair of *Scolopendra galapagoensis* just before mating **D** antennae tapping under the lifted ultimate legs in *Scolopendra dehaani* in courtship behaviour.

Ritualised meeting reactions have been described for *Scolopendra cingulata* Latreille, 1829 (Klingel 1960), *Cormocephalus anceps anceps* Porat, 1871 (Brunhuber 1969) and observed in *Scolopendra galapagoensis* Bollman, 1899 (Kronmüller 2010, unpublished data). When two specimens meet, each attempts to grasp the posterior region of the trunk of the other with its last pair of legs (Figure 2C).

In head to head contact both rapidly swing round the posterior part of body and attempts to grip the other with the ultimate, followed by the more posterior legs. Brunhuber’s plate 1A shows that the legs are parted and hooked over the body of the other animal. In this reaction the median spines on the ultimate legs seem to aid the grip. Kronmüller (unpublished data) observed that when two *Scolopendra dehaani* meet and they grasp each other in the previous described way, they hold this position for several minutes and up to more than half an hour. This ritual may be to avoid any aggressive behaviour.

A short video clip of this meeting reaction can also be seen on the website http://www.scolopendromorpha.com.

**Courtship behaviour**

During courtship behaviour in *Cormocephalus anceps anceps* the “defence posture” is adopted both partners use their antennae to tap the posterior part of the body especially the ultimate legs of the opposite sex (Figure 2D). Tapping and/or stroking with the ultimate legs is carried out by both sexes. The ultimate legs are waved sideways and tapped irregularly up and down and quite quickly (Brunhuber 1969) [for a recent review see Rosenberg (2009)].

**Species recognition**

Lewis (1985) argued that as the prefemoral spines on the ultimate legs are directed backwards (posteriorly) it is unlikely that they are used to facilitate gripping for which they should be directed forwards (anteriorly). He suggested that the function of the spines was that of specific discrimination during courtship prior to sperm transfer. If this were the case then one would not expect sympatric species to have a similar spine arrangement. There followed a lengthy discussion to justify this hypothesis. However no evidence has been found in this investigation to support this suggestion.

## Laboratory experiments

### Material and methods

Laboratory experiments were carried out to further investigate behaviour in prey capture as well as possible defence reactions in case of a predator attack. To simulate a the attack of mammal or bird predator, a piece of foam held with 25cm forceps was used to tap the centipedes from above.

The centipedes that were used for the experiments were either adult wild caught specimens (*Scolopendra heros*, *Scolopendra subspinipes*, *Ethmostigmus trigonopodus*, *Scolopendra morsitans*, *Scolopendra hainanum*, *Cormocephalus aurantiipes*) or, in one case, an adult captive bred specimen (*Scolopendra galapagoensis*).

The specimens were kept for at least 12 weeks before the experiments started in plastic containers of different sizes depending on the size of the specimen, with an air temperature of 25 degrees Celsius in day-time (around 10 hours illumination with a neon lamp) and 20–22 degrees Celsius at night-time. The average air humidity was around 70%. All plastic containers had a layer of humus and a piece of bark as a hiding-place. Once a week, the humus was sprayed with water to keep it moist and the specimen was fed one adult cricket (*Gryllus assimilis*).

For the experiments, the centipedes were moved to a 20 × 20 cm plastic container with a layer of about 2cm humus and left for at least one hour without disturbance before the experiments started. After each experiment, the centipedes were left for a further hour to recover before the next experiment.

The first experiment involved tapping the centipedes at different parts along the length of the body (anterior third, middle third and posterior third) to observe possible differences in defense reactions and to compare the responses of the investigated species.

In a second experiment a piece of bark was placed in the container as a refuge and the layer of humus was increased to 10cm. A centipede was placed in the container and as soon as the first third of the trunk was hidden either by the centipede crawling under the bark or by it digging into the humus, the tapping experiments described above were repeated.

Photos and short video clips are made with a Canon EOS 60D and a Canon EOS 6D, connected either to a Canon standard lens (18-55mm) or a Canon macro lens (100mm), three Canon Speedlight EX550/EX430 flashes (photos only), a tripod and a remote control (video clips).

### Results of the laboratory experiments

Initial experiments were carried out on an adult *Ethmostigmus trigonopodus* (Leach, 1817) from Tanzania, length 13 cm. When tapped on the first third of the trunk (near the head), the centipede attacked the forceps with the forcipules. When tapped on the posterior third, or even at the ultimate legs, it adopted the warning position, raising the ultimate legs to display the ventral and medial prefemoral spines as well as the coxopleural processes which are also equipped with spines. In the most cases the centipede directed the warning position towards the forceps by turning the posterior segments. In some cases, depending on the strength and speed of the simulated attack, the centipede counter-attacked the forceps with the claws of the ultimate legs by chopping down on them after lifting them high into the warning position.

When the *Ethmostigmus trigonopodus* was tapped in the mid part of the trunk, the centipede curled sideways to reach the forceps with the forcipules and the ultimate legs simultaneously. These observations were repeated in experiments with Scolopendra heros var. arizonensis Kraepelin, 1903 (USA), *Scolopendra galapagoensis* (Peru), *Scolopendra morsitans* (Pakistan), *Scolopendra cingulata* (Spain), *Scolopendra subspinipes* (Indonesia), *Scolopendra hainanum* Kronmüller, 2012 (China), *Scolopendra spinosissima* Kraepelin, 1903 (Philippines), and *Cormocephalus aurantiipes* (Newport, 1844) (Australia). All showed the same warning posture raising the ultimate legs (Figure 2A).

In addition *Scolopendra galapagoensis* lifted not only the ultimate legs into the warning position but also the last 3-4 pairs of locomotory legs (Figure 2B). This may be to present the distodorsal prefemoral spines on these legs. This behaviour was not seen in Scolopendra heros var. arizonensis, the other New World species investigated which lacks these spines.

In the experiments with bark and a thicker layer of humus, no difference was observed in the behaviour depending on whether the specimen was tapped on the mid part of the trunk or on the last third. The result was the same when the centipede was fed (*Schistocerca* sp.) and the forcipules as well as the anterior pairs of locomotory legs were involved in feeding. In this situation the centipede could not use the forcipules to carry out a warning bite and the only defence behaviour was hitting with the ultimate legs and stabbing with the ultimate leg pretarsi (claws). The experiment was repeated with *Ethmostigmus trigonopodus*, *Scolopendra galapagoensis*, *Cormocephalus aurantiipes* and *Scolopendra subspinipes*, which all showed the same results.

## Discussion

The scolopendrid species investigated here have unmodified and relatively robust ultimate legs. Species with very long thin ultimate legs such as some Otostigmus (Otostigmus) and *Rhysida* species, have not been investigated.

Bücherl (1971) stated that “The specimens of *Scolopendra* and *Otostigmus*, *Cryptops* and others attack their prey with the last prehensorial anal legs, then the head is rapidly curved behind and the venom claws deeply and firmly buried in the body of the prey.” Lewis (2010) concluded that there was no evidence to support the suggestion that the ultimate legs of *Cryptops* were involved in the capture of prey, instead suggesting that they are defensive; trapping some part of a potential predator and then being autotomised as the centipede makes good its escape. Dugon and Arthur (2012) stated that several species of scolopendrids had been observed seizing prey with the terminal pair of legs and then flicking the anterior half of the body to inject the prey with venom but in experiments with *Scolopendra subspinipes mutilans* Koch, 1878 (now *Scolopendra subspinipes* Leach, 1815) prey was only attacked with the forcipules.

We conclude that in the case of the species investigated the use of the ultimate legs is primarily for defence, however, should potential prey make contact with the centipede, the defensive response and defensive biting will lead to feeding whereas in the case of a predator, the centipede will try to escape after one or more warning bites.

Rowland Shelley (email dated 03 January 2011) notes “that there are frequently spines on the medial surface of the scolopendrid (ultimate leg) prefemora and I’ve always presumed that these must function to hold something steady I had always thought this would be prey... ” We were unable to confirm that scolopendrid centipedes hold prey or predators between the ultimate leg prefemoral, although in the ritualised meeting reaction in some species, the median spines on the ultimate legs seem to aid the grip.

The four main functions in species in which the ultimate legs are little specialised are:

1. Acting as hooks to suspend the animal for example from cave ceilings.
2. Warning position, when the legs are raised and splayed to reveal the prefemoral and coxopleural spines.
3. Stabbing as a defence, especially when the front part of the trunk and the head are unable to carry out any defensive action.
4. Grasping, the legs being flexed as when two animals assume the defence posture or as part of the mating ritual. In this case posteriorly directed spines would aid the grip. No evidence was found of objects being held between the prefemora.

The investigated centipedes (*Scolopendra heros*, *Scolopendra subspinipes*, *Scolopendra morsitans*, *Scolopendra hainanum*, *Scolopendra galapagoensis*, *Scolopendra spinosissima*, *Cormocephalus aurantiipes*, *Ethmostigmus trigonopodus*) are from different genera, different continents and different types of habitat. Nevertheless, their behaviour in prey capture and defense reaction do not differ. Also the meeting reaction seems to be very similar the same in all observed species.

In addition to the behavioural functions of the ultimate legs, two visual signals should be considered:

### Auto mimicry

**Figure 3.**
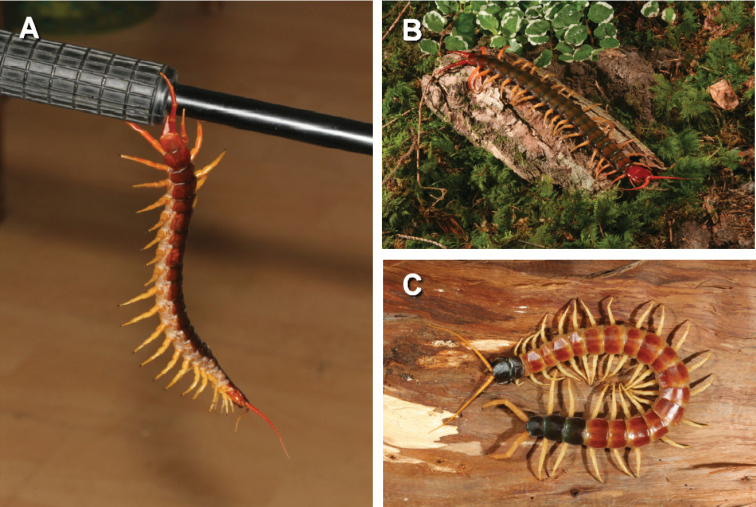
**A** *Scolopendra dehaani* hanging from a camera tripod just using the ultimate legs **B** *Scolopendra multidens* and **C** *Scolopendra heros* showing possible auto mimicry as the ultimate legs and last segments of the trunk mimic the head and antennae.

Some scolopendrid centipedes, especially the giant *Scolopendra* sp. have striking colour patterns. In several species, the colouration of the last trunk segments and the ultimate legs is the same as the colouration of the head and antennae (Figure 3 B and C). Predators may not be able to differentiate between the head and the posterior part of the trunk.

### Possible signal co-evolution

The warning posture, especially that of *Scolopendra galapagoensis*, where the last three or four pairs of locomotory legs are raised in addition to the ultimate pair, resembles that of spiders such as *Phoneutria* sp. or tarantulas and may be a case of possible signal co-evolution.
